# Medical Students’ Development of Ethical Judgment – Exploring the Learners’ Perspectives using a mixed methods approach

**DOI:** 10.3205/zma001073

**Published:** 2016-11-15

**Authors:** Thorsten Langer, Danny Jazmati, Ole Jung, Christian Schulz, Martin W. Schnell

**Affiliations:** 1 University Children's Hospital Freiburg, Division of Neuropediatrics and Muscle Disorders, Freiburg, Germany; 2 Harvard Medical School, Boston Children’s Hospital, Institute for Professionalism and Ethical Practice, Boston, USA; 3 Witten/Herdecke University, Institute for Ethics and Communication in Healthcare, Witten, Germany; 4 King’s College London, Institute of Psychiatry and Neuroscience (IoPPN), Maudsley Training Programme, London, UK

**Keywords:** medical ethics, teaching, students, mixed methods

## Abstract

**Objective: **Contemporary healthcare requires physicians to have well developed ethical judgment skills in addition to excellent clinical skills. However, no consensus has been reached on how to best teach ethical judgment skills during medical training. Previous studies revealed inconclusive results and applied varying theoretical frameworks. To date, the students’ perspectives on their development in ethical judgment has received less attention. Better insights in the learners’ experiences can help to improve educational interventions in medical ethics.

**Methods: **A vignette featuring a challenging case with opposing views between a patient’s parents and a physician followed by a questionnaire was presented to a cohort of medical students at a German medical school at three points in time during their medical training (Year 1, 2 and 5). The questionnaire included closed and open-ended questions addressing the participant’s preferred, hypothetical actions, their reasoning as well as the resources informing their reasoning. Content analysis was used for qualitative data; frequencies and percentages were used to describe quantitative findings.

**Results: **The response rate remained stable (28%) over the study period. Participants’ responses changed overtime. Accepting parents’ autonomy in the decision-making process was the majority standpoint of students in year 1 and 2 and became less often cited in year 5 (Year 1/2/5: 68/67/48%). On the contrary, not readily following the parents’ decision for medical reasons was a minority standpoint in year 1 and became more prevalent over time (year 1/2/5: 12/17/42%). Judgments were only partly based on ethics training. Instead, participants drew on experiences from their clinical clerkships and their personal lives. Throughout the study, participants did not feel well-prepared to make a judgment in the case (Average 2.7 on a Likert-Scale; 1=very well prepared, 4=very poor).

**Conclusions: **Over the course of their medical training, the participants seemed to increasingly frame the presented vignette as a medical problem. To optimize the development of ethical judgment teaching of ethics should be more integrated in clinical teaching. In addition to the analysis of rare and extreme cases, teaching ethics should also expand on challenges students and junior doctors commonly encounter themselves to promote ethical sensitivity and confidence in students.

## Zusammenfassung

**Hintergrund: **Die gegenwärtige Medizin erfordert von Ärzten nicht nur exzellente klinische Fähigkeiten, sondern auch ein hochentwickeltes, ethisches Urteilsvermögen. Bisher gibt es jedoch keinen Konsens darüber, wie man ethisches Urteilsvermögen während des Medizinstudiums optimal vermittelt. Vorangegangene Studien erbrachten keine eindeutigen Ergebnisse und verwendeten uneinheitliche theoretische Konzepte. Insbesondere die Perspektive der Studierende auf die Entwicklung ihres ethischen Urteilsvermögens erhielt wenig Aufmerksamkeit. Einsichten in die Lernerfahrungen der Studenten können helfen, die Entwicklung der ethischen Urteilsfähigkeit besser zu fördern.

**Methoden: **Den Teilnehmern wurde dreimal während des Studiums (1., 2. und 5. Jahr) eine Vignette vorgelegt, in der ein komplexer Fall beschrieben wird und in der die beteiligten Eltern des Patienten und der Arzt unterschiedliche Perspektiven einnehmen. Der begleitende Fragebogen beinhaltete geschlossene und offene Fragen, die das hypothetische Vorgehen des Teilnehmers, seine Begründung und die hinzugezogenen Ressourcen explorieren. Eine Inhaltsanalyse wurde für die qualitativen Daten verwendet; absolute Häufigkeiten und Prozentangaben wurden bestimmt, um quantitative Untersuchungsergebnisse zu beschreiben.

**Ergebnisse: **Die Rücklaufquote blieb über den Untersuchungszeitraum stabil (28 %). Bei den Antworten zeigten sich Veränderungen im Untersuchungszeitraum. Im ersten und zweiten Jahr vertrat die Mehrheit der Teilnehmer den Standpunkt, dass die Autonomie der Eltern im Entscheidungsfindungsprozess Vorrang habe, was im 5. Jahr seltener angegeben wurde (Jahr 1/2/5: 68/67/48%). Im Gegensatz dazu vertraten im Jahr 1 wenige Teilnehmer den Standpunkt, dass dem Wunsch der Eltern aus medizinischen Gründen nicht zu folgen sei; eine Haltung die später häufiger eingenommen wurde (Jahr 1/2/5: 12/17/42%). Der Ethikunterricht hatte in der Urteilsfindung eine mäßige Bedeutung. Vielmehr beriefen sich die Teilnehmer auf Erfahrungen aus ihrem klinischen Praktikum und ihrem Privatleben. Zu allen Erhebungszeitpunkten fühlten die Studenten sich nicht gut vorbereitet, eine Beurteilung in dem Fall abzugeben (Durchschnitt von 2,7 auf einer Likert-Skala; 1=sehr gut vorbereitet, 4=sehr schlecht vorbereitet).

**Zusammenfassung: **Im Verlauf des Medizinstudiums ordneten die Teilnehmer die in der Vignette dargestellte Entscheidungssituation zunehmend als ein medizinisches Problem ein. Um die Entwicklung des ethischen Urteilsvermögens zu unterstützen, sollte die Ethiklehre vermehrt in die klinische Ausbildung integriert werden. Zusätzlich zur Analyse seltener und extremer Fälle sollte die Ethiklehre Situationen und Herausforderungen thematisieren, die Studierende und junge Ärzte häufig antreffen, um ethische Sensibilität und Selbstvertrauen zu fördern.

## 1. Background

In modern healthcare clinicians frequently encounter situations in which they require not only good clinical but also ethical judgment. These demands have been widely acknowledged and led to the development of ethics curricula in many countries since the 1970s in order to provide future doctors with the skills to manage such situations [1], [2], [3]. In Germany, the discipline of medical ethics became a mandatory component of the undergraduate curriculum by implementation of the Medical Licensure Act in 2002, while pilot programs on the individual institutional level had been preexisting [4] including case-based seminars which in some universities had been by developed medical students themselves [5]. In order to comply with those regulations, German medical schools are obliged to offer education and summative assessment of students’ performances combining the disciplines of ethics, history and theory of medicine [6].

Parallel with the launching of medical ethics curricula research in how medical students actually develop ethical reasoning skills began to evolve [7]. In the past 30 years, numerous studies have been conducted with varying approaches and goals. One important line of research is to determine the outcome of a teaching intervention with regard to the increase of knowledge and ethical reasoning skills [8], [9]. As an example, Goldie et al. compared the judgment made by medical students in nine given ethical dilemmas with reference standards represented by an existing consensus from a professional institution and found that participants improved [10]. 

Another influential line of inquiry is to determine the impact of the overall educational experience on the moral development of medical students. In several studies the theory of moral development by Lawrence Kohlberg was applied for this end. Kohlberg’s theory suggests a given set of six subsequent stages of moral development that a person runs through from early childhood to adulthood. Studies within this developmental framework also use vignettes in which the structure of the answers can be matched with one of the stages of moral development. It has been shown that moral development in medical students occurs in a different way compared to the general population, i.e., they do not proceed to a higher level over time but stay on the same or even regress to a lower level [11], [12], [13], [14], [15]. 

To date, the perspective of medical students who are taught medical ethics has received less attention and has mostly been studied through cross-sectional surveys. Johnston et al asked medical students in the UK about their general views on teaching ethics and law, and found they perceived it as relevant [16]. A survey among German medical students showed that participants considered medical ethics an important subject [17]. However, in a cross-sectional study among medical students from Munich, Germany the authors found that respondents had minor levels of knowledge on various topics related to ethics, theory and history of medicine [18]. Although these results are encouraging for educators in medical ethics and indicate substantial room for improvement, we found no studies investigating the learners’ experience in the application of medical ethics in a clinical context. However, a better understanding of how medical students’ integrate ethical and clinical reasoning skills over time and how they experience their ethics training can help to better tailor the educational activities to the learners’ needs and to ultimately achieve better outcomes.

To address this gap we investigate four research questions in this study:

1. How does the judgment of a cohort of medical students in a case vignette develop over the course of their medical training?
2. How does the justification of their position change over time?
3. What resources do medical students draw on when they make their judgment?
4. How well do medical students feel prepared to make a judgment in a clinical vignette?

## 2. Methods

### 2.1 Design

We conducted a longitudinal study using a clinical vignette applying qualitative and quantitative methods. Students were repeatedly presented a clinical vignette in year 1, 2 and 5 of their training at the medical school of Witten/Herdecke University in Germany. The independent ethics committee at Witten/Herdecke University approved the study (Ref: 129/2013). 

#### 2.2 Setting

The medical school at Witten/Herdecke University was one of the first medical schools in Germany to introduce a longitudinal ethics curriculum in 1999. Medical training in Germany takes six years of which medical students their last year almost entirely in a hospital setting. Medical ethics at Witten/Herdecke University is being taught in the form of lectures, small-group learning and role play. It runs from semester one through nine with a total volume of 12x90 minutes. Learners’ progress is assessed by written essays, oral presentations and written exams. Regarding the content being taught the following themes are included: patient autonomy, benevolence, legal principles, guardianship and others [4], [19], [20], [21].

Among medical schools in Germany, Witten/Herdecke University has long been in a special role as it was the first approved to have a reform curriculum. Reform elements included a curriculum based on principles of problem-based learning (PBL), an integration of pre-clinical and clinical contents and a strong emphasis on primary care and patient-orientation. The number of students enrolled per year (n=84) is lower than in most other medical schools and students have to pay tuition. In recent years, several medical schools in Germany have introduced PBL curricula and other elements of curriculum reform. 

#### 2.3 Recruitment

The study was presented during the opening seminar of each particular year and students were invited to participate (n=84). Participation was voluntary and participants received no incentive. The case and a questionnaire were handed out with a stamped and addressed envelope. In order to increase returns two follow-up email reminders were sent out. Participation was anonymous. 

#### 2.4 Vignette and questionnaire

**Figure 1:**
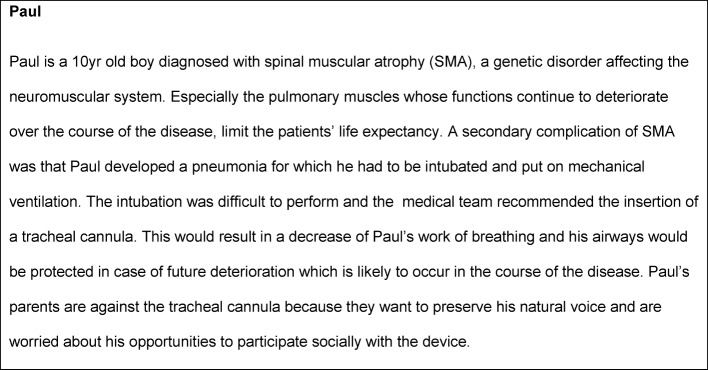
Shortened version of the vignette presented to participants (the full version is available in the attachment)

The vignette was developed by an interdisciplinary team involving collaborators from general pediatrics, pediatric intensive care, medical ethics and nursing science [22], [23]. A shortened version is shown in Figure 1 (Fig. 1) (full version see attachment ). 

The questionnaire was developed by the same interdisciplinary team. It consists of six open ended and five closed questions focusing on the role the doctor should take in decision-making process (e.g., should the doctor accept the parents’ decision (yes/no)? Please, explain your position.) Additional questions addressed the participants’ experience when answering the question. (e.g., what helped to reach an answer, what was difficult?) Vignette and questionnaire were pilot-tested by a sample of four medical students [24]. (The full vignette and the questionnaire are accessible as additional files 1 and 2 in German and in an English translation.)

#### 2.5 Analysis

Qualitative data from open ended questions were electronically transcribed. We then performed a content analysis [25]. The goal of the analysis was to identify recurring aspects in the students’ accounts in order to reconstruct the students’ perspective as a “co-ordinated set of ideas (…) a person uses in dealing with a problematic situation” [26]. Thereby, we aimed to summarize the participants’ accounts and to reconstruct the underlying concepts informing their answers.

TL and MWS independently coded data from all three collection points and discussed their results in a series of consensus meetings. Categories were refined in a circular process. Areas of disagreement between raters were examined and discussed until consensus was achieved. For the purpose of the presentation of data, TL and MWS chose quotes which represent a specific code particularly well.

In a second step, we determined the prevalence of coded themes in the data to allow insight into the predominance of certain thematic domains [27]. Therefore, each utterance was assigned a certain code and their relative frequencies were calculated. The goal of the integration of numbers in the qualitative analysis was to make statements which are often used in qualitative research, such as “some,” “usually,” and “most” more precise. However, it should be noted that the use of numbers does not imply the existence of correlations or causal relationships. 

The coding process was informed by a previous analysis after the first two points of data-collection (year 1 and 2) which focused on the way how participants describe Paul’s situation differently: In year 1, students described the situation predominantly through the perspective of Paul and his parents and referred to the ultimate outcomes of the disease (e.g., life vs. death). Participants’ descriptions in year 2 still incorporated Paul’s perspective but were also characterized by an increased use of medical terms (e.g. neurological impairment). The full results of this analysis are reported elsewhere [28]. 

Statistical analysis of the numerical data from closed questions was performed using SPSS 21.0 for Windows using frequencies and percentages. Due to the qualitative and hypothesis-generating design of the study no statistical significance tests were performed. 

## 3. Results

### 3.1 Participants

The rate of participation remained stable during the study (Year 1/25, Year 2/24, and Year 5/24). The response rate was 28%. Reasons for non-participation as elicited during seminars were perceived insufficient knowledge and/or pressure with exams. 

The following findings are presented in the order of the four research questions. 

#### 3.2 What should the doctor do?

**Figure 2:**
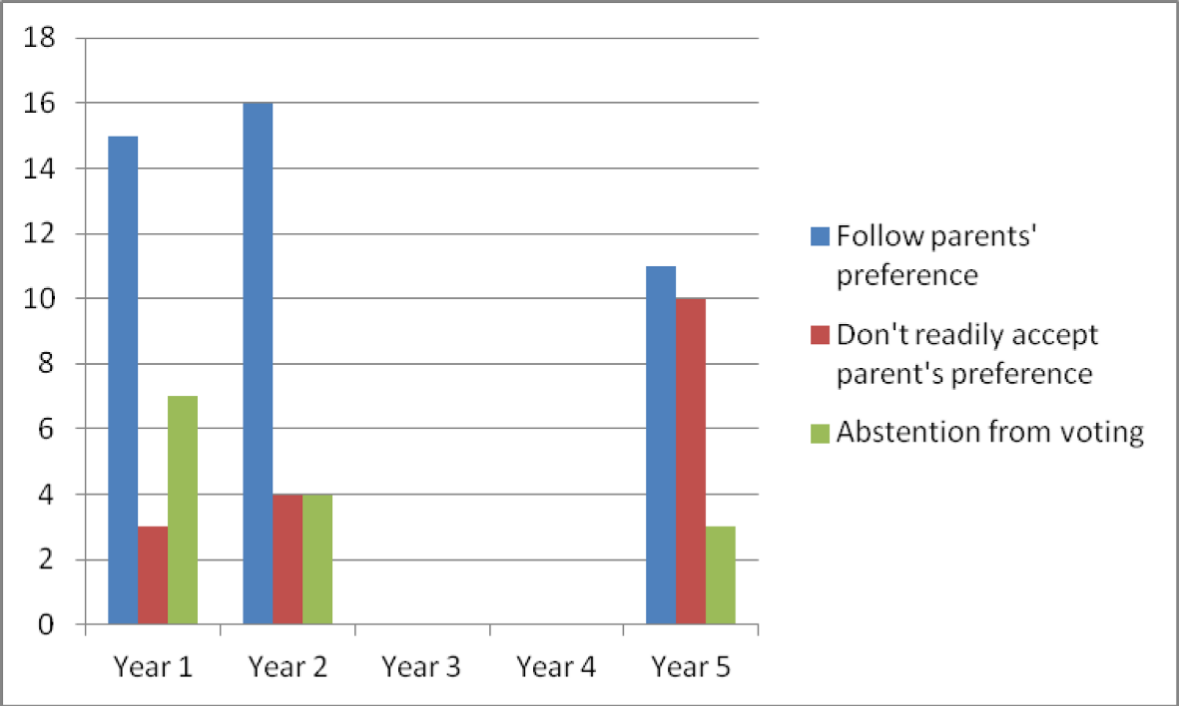
Distribution of participants’ positions regarding the doctor’s preferred action strategy (in absolute frequencies)

After reading the vignette, participants were asked what the doctor should do regarding the parents’ preference not to consent to the tracheostomy. In year 1, 68% of participants stated the doctor should accept the parents’ decision, 28% abstained from making a recommendation and 12% stated the doctor should not readily accept the parents’ decision (see Figure 2 (Fig. 2)).

In year 2, 67% of participants found that the doctor should accept the parents’ decision not to operate on Paul. The percentage of participants who abstained from voting decreased to 17%, and 16% of participants agreed with the view that the doctor should not readily accept the parents’ preference not to insert the tracheal cannula.

In year 5, fewer students (46%) agreed with the position to accept the parents’ vote. The group of participants absenting from a decision decreased further (13%) and the percentage of participants agreeing that the doctor should not readily accept the parents’ preference increased to 42%.

#### 3.3 Justification of the decision

The analysis of the open-ended questions revealed that participants voting to follow the parents’ preference took on two different perspectives to justify this position.

- Legal context: *“As Paul’s parents are his legal guardians their ultimate decision must be respected.”*
- Paul’s parents as his surrogate: *“His parents should decide, because they know him best. They decide in his best interest. The doctor is the adviser and companion in this situation – no matter, how the parents decide.”*

Participants taking on the opposite position applied the following perspectives:

- The tracheal cannula as medical necessity: *“Not to operate puts the child’s health at risk and the doctor should decide in Paul’s best interest to protect his life.”*
- Doctor as Paul’s advocate: *“The parents’ opinion doesn’t necessarily reflect Paul’s preference. The doctor should help Paul come to a decision himself.”*

**Figure 3:**
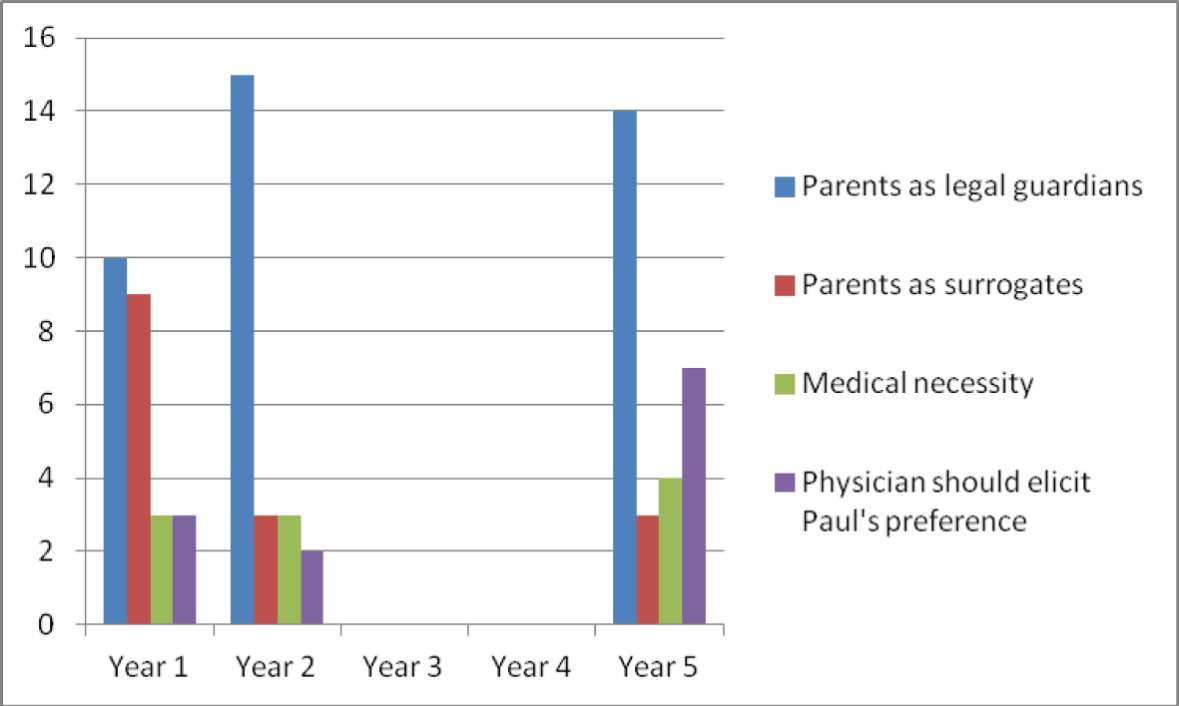
Distribution of participants’ arguments to justify their position (in absolute frequencies)

The quantitative distribution of these positions is displayed in Figure 3 (Fig. 3). The majority of participants over the course of the study cite the parents’ role as legal guardian which puts them in a position to make the decision on Paul’s behalf. The perspective that the parents are Paul’s surrogate is most frequently applied in year 1, drops remarkably in year 2 and remains at a lower level in year 5. The position which frames the decision as a medical necessity is cited by a smallest group of participants throughout the study. Finally, the perspective that the doctor should help Paul to be included in the decision-making process is applied by more participants in year 5 than any other year. 

#### 3.4 Resources informing participants‘ judgment

**Figure 4:**
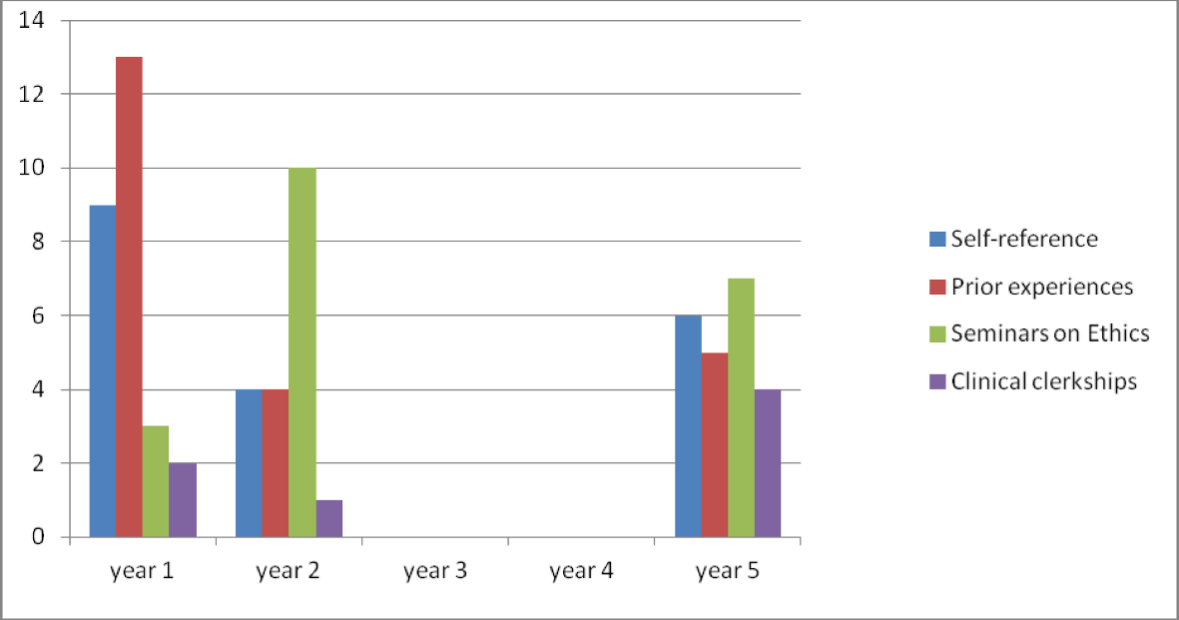
Resources participants draw on to make their judgment (in absolute frequencies)

The resources the participating students reported to draw on in answering the questionnaires are shown in Figure 4 (Fig. 4). In year 1, the vast majority indicated that either self-referential resources or personal/professional experiences prior to medical school informed their judgment. The category “self-reference” comprises areas that do not relate to any other area than the student himself/herself without further elaboration, e.g. “my conscience” or “my own ethical values”. In the category of personal and professional experiences participants had indicated that e.g., “caring for a family member with a similar condition” helped them to make a judgment. As expected, the amount of statements relating to seminars and courses at the medical school are considerably small in year 1.

The proportion of participants reporting to draw from their educational experiences in medical ethics increased in year 2, seemingly at the expense of prior experiences and self-reference, whereas the contribution of clerkship experience is still small.

Compared to the earlier points of data collection more students mention clinical clerkships to inform their judgment in year 5. At this point, none of the 4 resources can clearly be labelled superior to the others in its influence.

#### 3.5 Participants’ subjective preparedness for the case

Participants were asked to indicate on a Likert Scale “how well prepared you feel to make a judgment in a case like Paul’s” (1=very good, 4=very poor). Besides all the changes in the other outcomes, the subjective preparedness to make a judgment remained stable at a less than good level over the three points of data collection (see Table 1 (Tab. 1)).

One item in the questionnaire addressed the role of an ethics board. The vast majority of respondents voted to consult a clinical ethics board in this case at all three points of data collection (92% year 1,80% year 2,96% year 5). 

## 4. Discussion

This study describes the development of the ethical judgment in a cohort of German medical students using a vignette. It further reports on the participants’ resources and their subjective preparedness to make a judgment in the hypothetical case.

### 4.1 Interpreting changes over time

We found remarkable changes in the participants’ responses over time. With regard to the doctor’s role in the decision making process most participants stated the doctor should follow the parents’ preference throughout the study. However, in year 5 less participants abstained from making a statement at all and more voted the doctor should not readily accept the parents’ decision compared to the earlier stages of the study. This can be described as a change in the view on the doctors’ role over time. The doctor becomes increasingly involved in the decision making process and takes on more responsibility. “Not readily accepting the parents’ decision” means that he will engage the family in a discussion and explain his point of view. The notion of a more active role can be supported by the finding that more respondents ask for the child’s preference in year 5 what implies the doctor interferes with the relationships within the family.

The observation of a changed role of the doctor towards the end of medical school leads to two possible interpretations – a more favorable and a more critical one. First, the increased percentage of respondents not readily accepting the decisions shows that medical students become aware of their responsibility they have for their patients which exceeds the mere exchange of information and viewpoints but also involves discussions with parents and patients, especially when their viewpoint differs from the one parents hold. Within the preserved boundaries of the patient’s and parents’ autonomy this would be a development many educators would probably welcome. Second, the stronger tendency not to readily accept the parents’ decision could reflect that medical students increasingly see themselves as more knowledgeable than parents to make a decision based on their medical background. In addition, the increased medical knowledge base could lead students to “re-frame” the issue from an ethical to a medical decision and thereby further devaluing the parents’ contribution. The latter interpretation would mean to override parent’s autonomy and therefore would represent a result not at all intended by most ethicists. The present study does not provide sufficient data to favor one or the other interpretation and maybe both hold true depending on the individual student. However, for teaching purposes both interpretations may be useful to facilitate a reflective discussion with students in a seminar.

#### 4.2 Participants’ resources to make an ethical judgment

With regards to the resources respondents drew on, seminars in ethics and clinical clerkships helped participants to make their judgment in addition to their prior experiences and their self-referential resources. However, there are two concerns associated with this finding. First, does the development described above reflect the intentions and goals of an ethics curriculum? The goal of teaching ethics at our institution – as probably in many others - is process-oriented instead of “providing ready solutions to difficult cases”. Medical students are encouraged to think in a reflective way, be able to change perspectives, apply ethical principles and thus come to good decisions [4], [19]. This study raises some questions about the actual impact the teaching of ethics can have when competing with various other influences students are exposed to at medical school. Second, although participants drew on their learning experience from medical ethics, their subjective preparedness to make a judgment in this case remained stable and less than good throughout medical school. This raises the question, how medical students can possibly be better prepared for the ethical challenges in their professional lives? A discussion among curriculum planners from different fields and disciplines seems important to find ways how medical students can be supported and prepared for the challenges associated with their professional development which will most likely involve ethical conflicts.

#### 4.3 Implications for teaching medical ethics to students

Our findings resonate with other studies which showed changes in moral reasoning during medical training. Some authors who applied the theory of moral development by Lawrence Kohlberg reported regression of moral reasoning skills in medical students [13], [14], [15], others an arrest [29], [30]. As our study described the participants’ development in qualitative terms instead of applying an evaluative framework, it is difficult to compare the results directly. However, on the level of conclusion the studies point to the same direction: First, teaching ethics is has not reached its goals yet. Second, teaching ethics requires learners to truly engage in a reflective process. For these ends, most institutions apply case-based discussions instead of lectures as a teaching method [5], [11]. However, Truog et al. recently highlighted an important downside of the seemingly common approach to focus on extreme and controversial cases which are particularly suitable to demonstrate ethical principles. The authors argue that by focusing on the unusual cases the importance of the countless ethical decisions embedded in everyday clinical practice easily get neglected [31]. As an example, the question, “doctor, what would you do if this was your child?” could well be asked by Paul’s parents in our scenario and discussed in an ethics seminar [32]. Thus, the scope of teaching ethics could be widened to include the rare problems as well as the more common issues. 

Looking at our institution, this study will have specific implications for future teaching of ethics. Since the longitudinal ethics curriculum has been launched, considerable efforts have been taken to establish links to clinical cases and combined teaching formats with teachers from the clinical disciplines. These links should be reinforced to allow students to understand that medical and ethical challenges occur at the same time and interact with each other. Further, the students’ process of reasoning should be made more accessible in the teaching process. It should become clear to students that personal experiences can be an important resource for reasoning when applied in a reflected and cautious manner. This kind of learning experience requires a safe learning environment in which students can “open up” and discuss their own views with peers and faculty in a non-judgmental manner. Such an approach could enable learning as development and personal growth rather than an adoption of norms and content. In order to facilitate such learning, Dyche et al. ask educators to nurture their students’ curiosity [33]. As a contrast to the content-heavy parts of the medical curriculum which promote efficacy and passive learning, opportunities are essential in which students are encouraged to develop their own questions, “especially those who might withhold their questions for fear of appearing naïve”. The authors provide suggestions on how to promote such inquisitiveness and discovery among students in a teaching context. 

One way to facilitate such inquisitive learning is the use of student’s narratives. This approach has shown promising results to access the hidden curriculum [34], [35]. In his sentinel article introducing the concept of the hidden curriculum to medicine, Hafferty defines the hidden curriculum as “a set of influences that function at the level of organization structure and culture” [36]. By definition, these values are taught in an implicit way and they can affect a wide range of issues in the medical world. With regard to the case of patients with spinal muscular atrophy Type 1 for example, a survey among physicians from different disciplines has been conducted to elicit differences in practice. The authors found a wide variation in physician practice regarding the mechanical ventilation depending on their specialization (neurology, intensive care and rehabilitative medicine) [37]. Thus, this case seems like a perfect opportunity for an inquiry regarding the underlying, ethically relevant assumptions hold by practitioners – and students. However, the medical curriculum offers many more opportunities for students to develop questions of such kind and to make the hidden curriculum accessible to reflection and professional development [38]. The impact of such novel teaching strategies should of course be thoroughly evaluated.

#### 4.4 Limitations

This study has limitations. From a methodological viewpoint, a vignette study applied at three points in time during a 5-year curriculum offers a small window to explore the development of medical students’ judgment and other influences might be missed. Further, the use of one vignette at the three points of data collection could bias the response. We decided to use the same vignette to increase the level of standardization. However, a recall bias cannot be ruled out. The response rate was lower than expected. A possible bias due to non-reported results is possible.

## 5. Conclusion

This study explored the development of medical students’ ethical judgment as operationalized in the research questions mentioned above. The judgment changed over time but students felt less than well prepared throughout the study period. We therefore suggest that teaching ethics should be integrated even more into clinical training and should address more common ethical issues future doctors and even students may encounter in their everyday practice. 

## Authors’ contributions

TL and MWS conceptualized and designed the study. DJ and OJ collected and managed the data. TL, DJ, OJ, CS and MWS analyzed and interpreted the data. TL, CS and MWS drafted the first manuscript. All authors read and approved the final manuscript. 

## Acknowledgements

The authors would like to thank the medical students of Witten/Herdecke University for their participation. Manne Sjöstrand, MD PhD helped to improve the manuscript with his insightful comments. Finally, the authors want to thank Rita Fountain for her help in finalizing the manuscript.

## Funding

TL received grant support through the German Research Foundation (DFG) in 2013-2015 (LA 2344/2-1).

## Competing interests

The authors declare that they have no competing interests.

## Supplementary Material

Full version vignette presented to participants

## Figures and Tables

**Table 1:**
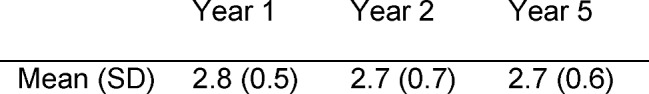
Subjective preparedness to make a judgment in this scenario (1=very good, 4= very poor)
