# Supplementary material for: Medical Students’ Development of Ethical Judgment – Exploring the Learners’ Perspectives using a mixed methods approach
Source: GMS J Med Educ. 2016 Nov 15;33(5):Doc74. doi: 10.3205/zma001073 (PMC5135414; doi:10.3205/zma001073)
Supplement: Full version vignette presented to participants [file JME-33-74-s-001.pdf]

Univ.-Prof. Dr. Martin W. Schnell, M.A.
Institut für Ethik und Kommunikation
im Gesundheitswesen
- Direktor -
Fakultät für Medizin
Private Universität Witten/Herdecke gGmbH
Alfred-Herrhausen-Straße 50
58448 Witten

Questionnaire

Medical students‘ development of the ethical judgment

Universität Witten Herdecke

Univ.-Prof. Dr. Martin W. Schnell, M.A.
Institut für Ethik und Kommunikation
im Gesundheitswesen
Private Universität Witten/Herdecke gGmbH
Alfred-Herrhausen-Straße 50
58448 Witten
schnell@uni-wh.de

Please indicate which semester you are currently studying:

1 ☐

2 ☐

3 ☐

4 ☐

5 ☐

6 ☐

7 ☐

8 ☐

9 ☐

10 ☐

1. Case Vignette

Paul is a 10 year old boy with spinal muscular atrophy (SMA). Until recently he was a 4th grade student in good standing. He was using a wheel chair and received assistance on his way to school and back. He needed help when he walked. He communicated without any problems. Coughing and mobilizing sputum is challenging. Therefore he receives physiotherapy. When having a cold, he occasionally uses a home-ventilator with a mask as he does not have enough strength to breath and mobilize the sputum.

Four weeks ago, Paul fell seriously ill. He got a high fever and dyspnoe. In the hospital a severe pneumonia was diagnosed. He was not able to breathe on his own, so he had to be intubated and connected with a ventilator. He received antibiotic treatment. The fever and inflammatory lab results normalized after one week. However, an attempt to wean Paul off the ventilator failed. After the extubation, Paul got exhausted after one hour and the pCO2 rised above 80mmHg. Paul had to be re-intubated and re-connected to the ventilator. Being on the ventilator, Paul cannot speak. He can communicate non-verbally.
In acknowledgment of the typical course of the SMA the medical staff does not expect that Paul will ever be strong enough to breathe through his mouth or nose without any assistance.

That's why the doctors suggest to operate on Paul and place a tracheostoma. This is an opening in the trachea below the larynx in which a plastic tube (cannula) will be inserted. Through this tube the patient is able to breathe with less effort as the airway resistance is lower. If a patient deteriorates in his respiratory function, a ventilator can be connected to the cannula.
By insertion of the tracheal cannula, a patient loses his/her original voice as the air does not flow through the vocal cords anymore. A speech cannula is a special cannula with which a patient can talk with an "artificial" voice with less expressive potential than the original voice.
Most patients with SMA keep the tracheostoma for their whole life.

Paul's parents do not wish a tracheostoma because they want to maintain Paul's possibilities of linguistic expression. They prefer another attempt to extubate Paul hoping he will be strong enough to breathe on his own. The doctors are very skeptical regarding this option and warn of the risks of an insufficient oxygen supply in an emergency re-intubation which could lead to long-term damage of his brain function. Pauls does not know the exact prognosis of his condition. No one has talked to him yet about a possible need for a tracheostoma and the possible loss of his original voice.

1. Should the doctor accept the parents‘ decision?

a) Yes, why:

b) No, why:

2. What consequences does the operation have? What consequences are to expected without an operation?

a) Consequences of the operation:

b) Consequences of not having an operation:

3. How should the parents be counselled?

a) ☐ Open-ended. The doctor should not voice his/her preference.

b) ☐ The doctor should voice his/her preference.

c) ☐ The doctor should get the youth welfare department involved.

Please explain your position:

4. How should the doctor communicate with Paul? (He is awake on the ventilator and can communicate visually and through written notes.)

a) ☐ He/she should advise the parents to talk to their child.

b) ☐ He/she should talk to the child – even if the parents do not wish for this.

c) ☐ He/she should not talk to the patient and continue to treat the patient medically.

d) ☐ Other suggestions, please explain:

5. Would you invite an ethics consult to discuss the case?

a) ☐Yes, because:

b) ☐No, because:

6. How well do you feel prepared to make a judgment in a case like Paul's?

a) ☐very well

a) ☐well

c) ☐poorly

d) ☐very poorly

7. What resources did help you in making a judgment in this case? Please, explain:

8. What was difficult in answering the questions? Please, explain:

Thank you very much!

Univ.-Prof. Dr. Martin W. Schnell, M.A.
Dr. med. Thorsten Langer
Ole Jung
